# High Amplitude Phase Resetting in *Rev-Erbα/Per1* Double Mutant Mice

**DOI:** 10.1371/journal.pone.0012540

**Published:** 2010-09-02

**Authors:** Corinne Jud, Antoinette Hayoz, Urs Albrecht

**Affiliations:** Department of Medicine, Unit of Biochemistry, University of Fribourg, Fribourg, Switzerland; Pennsylvania State University, United States of America

## Abstract

Over time, organisms developed various strategies to adapt to their environment. Circadian clocks are thought to have evolved to adjust to the predictable rhythms of the light-dark cycle caused by the rotation of the Earth around its own axis. The rhythms these clocks generate persist even in the absence of environmental cues with a period of about 24 hours. To tick in time, they continuously synchronize themselves to the prevailing photoperiod by appropriate phase shifts. In this study, we disrupted two molecular components of the mammalian circadian oscillator, *Rev-Erbα* and *Period1* (*Per1)*. We found that mice lacking these genes displayed robust circadian rhythms with significantly shorter periods under constant darkness conditions. Strikingly, they showed high amplitude resetting in response to a brief light pulse at the end of their subjective night phase, which is rare in mammals. Surprisingly, *Cry1*, a clock component not inducible by light in mammals, became slightly inducible in these mice. Taken together, *Rev-Erbα* and *Per1* may be part of a mechanism preventing drastic phase shifts in mammals.

## Introduction

In mammals, many physiological and behavioural parameters are subjected to daily variations. For instance, blood pressure, heart rate, body temperature, or sleep-wake cycles change considerably during the course of a day. The central circadian (Latin: “around a day”) clock, which governs these rhythms, is located in the suprachiasmatic nuclei (SCN) of the ventral hypothalamus [1], [2]. Since this internal pacemaker can tell time only approximately, it has to be reset every day by environmental signals. The strongest synchronizer for the endogenous clock is light, which signals directly to the SCN via the retinohypothalamic tract [3]. To reset peripheral oscillators, rhythms generated in the SCN are sent to the rest of the body via both neuronal and humoral signals.

Much of the understanding concerning the functioning of the circadian clock has come from extensive work done in model organisms mutant for one or several clock genes. In mammals, it was found that the molecular clockwork is based on cell-autonomous interlocked positive and negative transcriptional/translational feedback loop (TTL) [4]. The positive limb of the core loop is formed by the transcription factors CLOCK and BMAL1. Heterodimers of these proteins bind to E-box motifs in the promoters of *Per, Cry, Rev-Erbα* and other genes controlled by the clock. In the cytoplasm, the PER proteins form complexes with CRY, which then enter the nucleus. Once the complexes reach their destination, CRY inhibits the CLOCK/BMAL1 driven transcription, thereby establishing a negative feedback loop [5]–[7]. The suppressed E-box driven transcription leads to a decline in PER, CRY and REV-ERBα protein levels, which finally re-activates CLOCK-BMAL1 driven transcription. Due to the decrease in REV-ERBα, *Bmal1* transcription is no longer inhibited [8], [9]. Hence RORα can activate *Bmal1* expression by binding to the now vacant retinoic acid related orphan receptor response elements (RORE) in its promoter [10]–[12].

The TTL described above can be modulated by external signals. As mentioned previously, the strongest Zeitgeber (literally, “time giver” in German) for the mammalian clock is light. To better study the impact of light on the circadian system animals kept under constant darkness (DD) are subjected to a brief light pulse at different time points. During the subjective day, light has little or no effect on the resetting of the clock. By contrast, light exposure during the subjective night is capable of shifting the pacemaker. Depending on whether the light is administered during the early or late subjective night, the animal either delays or advances its clock, respectively [13]–[15].

Several studies attempted to identify the molecular mechanisms underlying resetting in response to a light pulse. However, in mammals only little is understood compared to *Drosophila* and zebrafish [16]–[18]. Since both *Rev-Erbα^−/−^* and *Per1^Brdm1^* single mutant mice are characterized by an aberrant light response during late night [9], [19], we were interested in analyzing the resetting phenotype of double mutants. We monitored their wheel-running behaviour under constant conditions and established a phase response curve (PRC). We show that *Rev-Erbα^−/−^Per1^Brdm1^* double mutant mice maintain a functional circadian clock although they display a shorter period with higher variance between individuals. Moreover, clock gene expression and their light induction are altered in mice deficient for both *Rev-Erbα* and *Per1*. Strikingly, they show high amplitude resetting and *Cry1* becomes slightly light inducible in these mice after a light pulse delivered at the end of the subjective night. We conclude that *Rev-Erbα* and *Per1* may participate in a mechanism leading to moderate light evoked behavioral responses typically observed in mammals.

## Results

### Generation of *Rev-Erbα^−/−^Per1^Brdm1^* double mutant mice

Since *Rev-Erbα* and *Per1* mutant mice both show shortened period length in constant darkness [9], [20], we wanted to test circadian parameters in mice bearing both mutations. To this end, *Rev-Erbα* [9] and *Per1* [20] single mutants were crossed. Double mutant offspring were intercrossed to produce both wild-type and mutant animals. Genotyping of these mice (Fig. S1) revealed that all genotypes were obtained at the expected Mendelian ratios (Fig. S2A). Moreover, they were all morphologically indistinguishable from their wild-type littermates. In addition, *Rev-Erbα^−/−^Per1^Brdm1^* double mutants were fertile and gave birth to normally sized litters at the expected intervals (Fig. S2B, S2C).

### *Rev-Erbα^−/−^Per1^Brdm1^* double mutant mice display a shorter circadian period in DD

**Figure 1:**
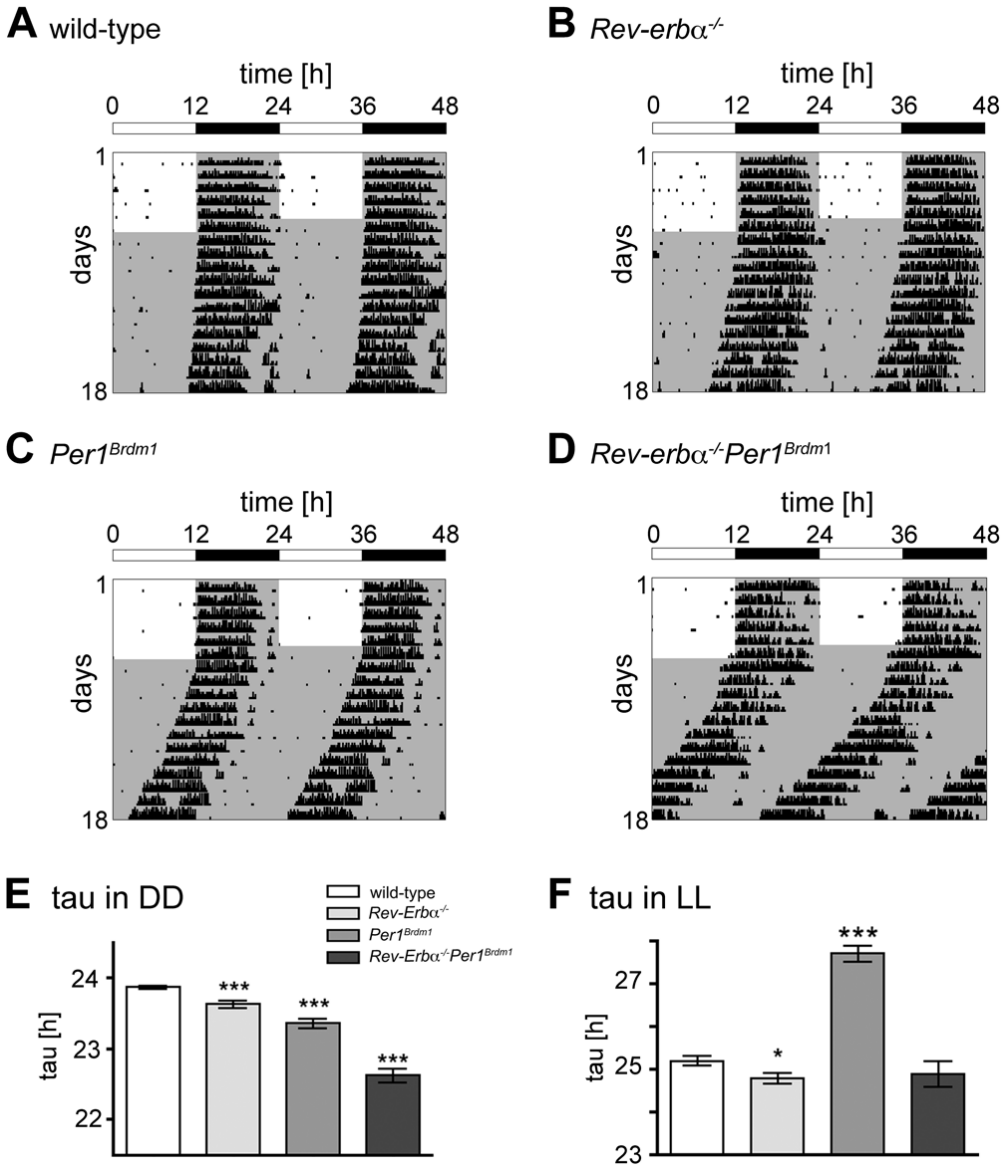
Representative wheel-running activity plots. (A–D) Representative locomotor activity records of wild-type (A), *Rev-Erbα^−/−^* (B), *Per1^Brdm1^* (C), and *Rev-Erbα^−/−^Per1^Brdm1^* double mutant mice (D) under both light/dark (white and grey shaded, respectively) and constant darkness (grey shaded) conditions. Black bars represent wheel-revolutions. The actograms are double-plotted with the activity of the following light/dark cycle plotted to the right and below the previous light/dark cycle. The white and black bar on the top of each actogram indicates light and darkness, respectively. (E) Average free-running period (tau) in constant darkness: *Rev-Erbα^−/−^* (23.62±0.05 h; N = 27), *Per1^Brdm1^* (23.35±0.06 h; N = 22), *Rev-Erbα^−/−^Per1^Brdm1^* mutant (22.62±0.1 h; N = 34), wild-type (23.86±0.02 h; N = 38). (F) Average free-running period (tau) in constant light. *Rev-Erbα^−/−^* (24.78±0.14 h; N = 8), *Per1^Brdm1^* (27.70±0.19 h; N = 7), *Rev-Erbα^−/−^Per1^Brdm1^* mutant (24.88±0.3 h; N = 11), wild-type (25.18±0.11 h; N = 12). Data are represented as mean ± SEM. Unpaired t-test was performed to compare the free-running period lengths. * p<0.05, *** p<0.0001 (compared to wild-type).

To study the effect of absent *Rev-Erbα* and *Per1* on locomotor activity, mice were housed individually and their wheel-running activity was monitored [21]. Initially, animals were kept in a 12-h light:12-h dark cycle (LD 12∶12 h) for several days until they were stably entrained. Subsequently, they were released into constant darkness (DD). Under LD conditions, *Rev-Erbα^−/−^Per1^Brdm1^* double mutants displayed an activity profile comparable to that of wild-type mice (Fig. S3). Upon release into DD, double mutants remained rhythmic and ran with a circadian period of 22.62±0.1 h (mean ± SEM; N = 34) which is significantly shorter than that of the wild-type (23.86±0.02 h; N = 38) and *Rev-Erbα* ^−/−^ (23.62±0.05 h; N = 27) or *Per1^Brdm1^* (23.35±0.06 h; N = 22) single mutant mice (Fig. 1A–E). This might indicate that the absence of these two clock genes has a cumulative effect on circadian period in DD.

Under constant light conditions, double mutant mice display a period, which is comparable to the one of wild-type and *Rev-Erbα* ^−/−^ animals (Fig. 1F). Interestingly, removal of *Rev-Erbα* in *Per1^Brdm1^* mutants normalizes the free-running period in LL to a level comparable to wild-type animals. Hence, it appears that knocking out both genes rescues the *Per1^Brdm1^* phenotype in LL.

### *Rev-Erbα^−/−^Per1^Brdm1^* double mutant mice display reduced amplitudes in clock gene expression

**Figure 2:**
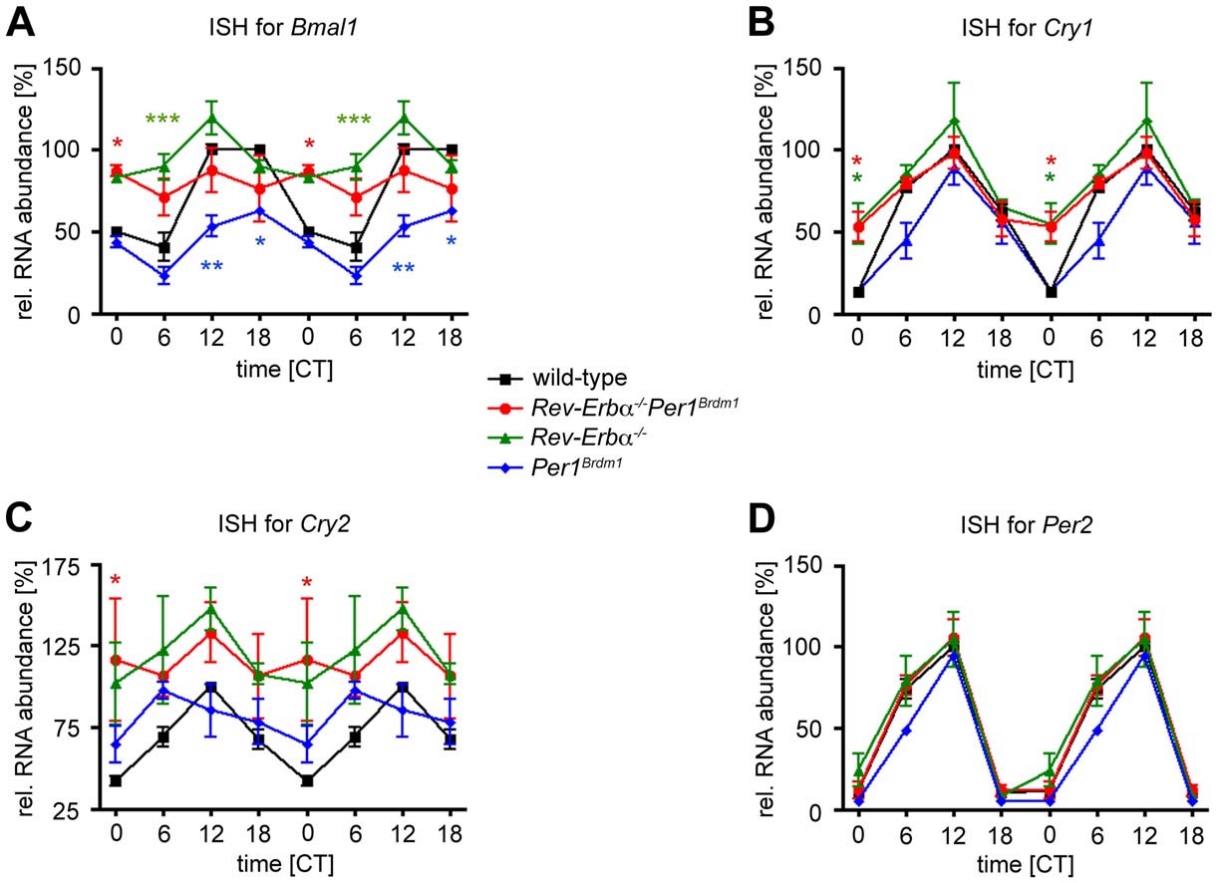
*In situ* hybridization (ISH) profiles of cycling clock gene expression in the SCN. Relative expression of *Bmal1* (A), *Cry1* (B), *Cry2* (C), and *Per2* (D) mRNA in the SCN under constant darkness conditions of wild-type (black line), *Rev-Erbα^−/−^Per1^Brdm1^* (red line), *Rev-Erbα^−/−^* (green line), and *Per1^Brdm1^* (blue line) mice. Values are double plotted and represented as mean ± SEM (N = 3). Two-way ANOVA with Bonferroni post-test was performed to determine significant differences between genotypes. * p<0.05; ** p<0.01; *** p<0.001.

*In situ* hybridization analysis was used to extend our investigations to the molecular level. Clock gene expression patterns in the SCN were determined on coronal brain sections of mice kept in DD for six days. *Bmal1* mRNA accumulation in *Rev-Erbα^−/−^Per1^Brdm1^* double mutant mice was constantly high (Fig. 2A). However, levels were slightly lower than those reported for *Rev-Erbα^−/−^* single mutants [9]. This in combination with the observation that the increase in *Bmal1* mRNA was significantly truncated in *Per1^Brdm1^* single mutant mice could indicate that PER1 may be involved in activating *Bmal1* expression.

The expression of *Cry1* was rhythmic with similar peak levels in all four genotypes (Fig. 2B). By contrast, the amplitude in *Cry1* mRNA accumulation was dampened in *Rev-Erbα^−/−^* single and *Rev-Erbα^−/−^Per1^Brdm1^* double mutant mice due to an elevated trough at circadian time 0 (CT0). Expression of *Cry2* mRNA was slightly higher in *Rev-Erbα^−/−^* single and *Rev-Erbα^−/−^Per1^Brdm1^* double mutant mice compared to *Per1^Brdm1^* single mutant and wild-type animals (Fig. 2C). However, these differences were only statistically significant at CT0 for the double mutant animals compared to the wild-type. Interestingly, disruption of *Per1*, *Rev-Erbα*, or both did not alter *Per2* expression patterns (Fig. 2D). Our observations confirmed the previous findings that disrupting *Rev-Erbα* considerably affected the expression of *Bmal1* and *Cry1* but had only little or no consequences on *Cry2* and *Per2* transcript levels [9]. While *Bmal1* expression seemed to be less changed in *Rev-Erbα^−/−^Per1^Brdm1^* double mutant mice compared to *Rev-Erbα^−/−^* single mutants, *Cry1* expression was unchanged between these two mutants.

With the exception of the *Per2* mRNA, overall clock gene expression patterns display lower amplitudes in *Rev-Erbα^−/−^Per1^Brdm1^* double mutant mice. This indicates that the oscillator in these animals is probably less robust (low amplitude), which might lead to high amplitude resetting (see below).

### *Rev-Erbα^−/−^Per1^Brdm1^* double mutant mice display high amplitude resetting

**Figure 3:**
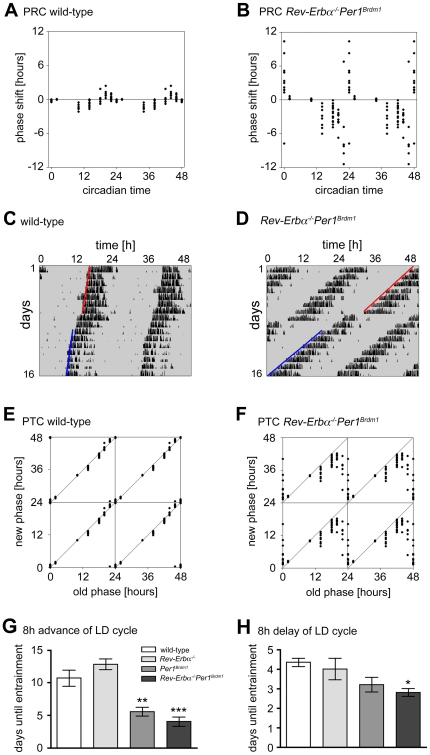
Phase shifting responses to light pulses and rescheduled light/dark cycles. (A/B) Phase response curve (PRC) of wild-type (A; N = 4–15) and *Rev-Erb^−/−^Per1^Brdm1^* (B; N = 8−15) double mutant mice to 15 min light pulses. The x-axis indicates the circadian time the light pulse was administered and the y-axis represents the phase shift produced by the light pulse. Each dot represents the phase shift of one animal. (C) Representative actogram of a phase-advance response to a light pulse at CT22 of a wild-type animal. (D) Representative actogram of a phase-advance response to a light pulse at CT22 of a *Rev-Erbα^−/−^Per1^Brdm1^* double mutant animal. Red lines: old phase, blue lines: new phase. (E/F) Phase transition curve (PTC) of wild-type (E) and *Rev-Erb^−/−^Per1^Brdm1^* (F) double mutant mice to 15 min light pulses. Data of A and B are replotted to obtain E and F, respectively. The x-axis indicates the circadian time the light pulse was administered (old phase). The y-axis displays the circadian time to which locomotor activity was shifted after the light pulse (new phase). (G) Average time to adjust to an 8-hour advance of the light/dark (LD) cycle. Data are represented as mean ± SEM. Unpaired t-test was performed to compare the days until re-entrainment of *Rev-Erbα^−/−^* (12.80±0.80 days; N = 5), *Per1^Brdm1^* (5.50±0.67 days; N = 6), and *Rev-Erbα^−/−^Per1^Brdm1^* (4.00±0.69; N = 7) mutant to wild-type (10.67±1.26 days; N = 6) mice. ** p<0.01; *** p<0.001. (H) Average time to adjust to an 8-hour delay of the light/dark (LD) cycle. Data are represented as mean ± SEM. Unpaired t-test was performed to compare the days until re-entrainment of *Rev-Erbα^−/−^* (4.00±0.55 days; N = 5), *Per1^Brdm1^* (3.20±0.37 days; N = 5), and *Rev-Erbα^−/−^Per1^Brdm1^* (2.80±0.20 days; N = 5) mutant to wild-type (4.33±0.21 days; N = 6) mice. * p<0.05.

The impact of the combined absence of *Rev-Erbα* and *Per1* on the clock-resetting response of mice was studied. A single 15 min light pulse was administered at various circadian times (CT) and the change of clock phase was quantified [22]. While wild-type mice displayed the expected low amplitude (Type I) phase response curve (PRC), *Rev-Erbα^−/−^Per1^Brdm1^* double mutants showed high amplitude (Type 0-like) resetting (Fig. 3A–D) [23], [24]. For double mutant animals, the so-called “breakpoint” (transition from phase delays to phase advances) was observed between CT22 and CT24. At this time point it was not possible to distinguish whether the animals make huge phase advances or delays. Interestingly, some mice became transiently arrhythmic after a light pulse given at CT22 for some days (data not shown). The difference between weak and strong resetting can be visualized more clearly when data are plotted as a phase transition curve (PTC). In this type of visualization, the time a light pulse was given is compared to the time the phase is shifted to after the light pulse (i.e. “old” *versus* “new phase”). In wild-type mice, all data points were found to be close to the diagonal of the square, which has a slope of 1 (Fig. 3E). Hence, the new phase was almost identical with the old one and they displayed a so-called Type 1 resetting. By contrast, the average slope for *Rev-Erbα^−/−^Per1^Brdm1^* double mutants deviated significantly from 1 (Fig. 3F).

Due to the strong resetting phenotype of the double mutant animals we were curious to see, how fast they would adapt to an experimental jet lag. Hence we entrained them to LD 12∶12 h before they were subjected to an 8 h advance of the lighting schedule. *Rev-Erbα^−/−^Per1^Brdm1^* double mutant mice achieved stable re-entrainment already after 4.00±0.69 days (mean ± SEM; N = 7) while it took two times longer for wild-type animals (10.67±1.26 days; N = 6; Fig. 3G). Likewise, *Rev-Erbα^−/−^Per1^Brdm1^* (2.80±0.20 days; N = 5) double mutants adapted faster to an 8 h delay in the photoschedule (Fig. 3H) compared to wild-type mice (4.33±0.21 days; N = 6).

### *Cry1* is slightly light inducible in *Rev-Erbα^−/−^Per1^Brdm1^* double mutant mice

**Figure 4:**
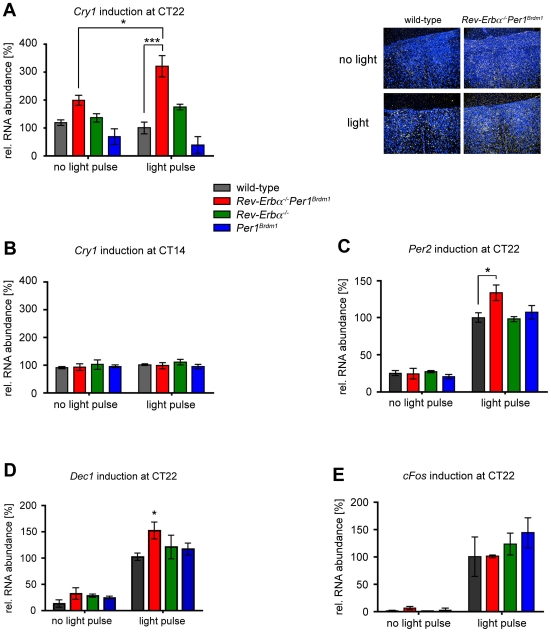
Light induced mRNA accumulation in the SCN. (A) *Cry1* induction after a light pulse at CT22 (N = 3); (B) *Cry1* induction after a light pulse at CT14 (N = 3); (C) *Per2* induction after a light pulse at CT22 (N = 3−5); (D) *Dec1* induction after a light pulse at CT22 (N = 3); (E) *cFos* induction after a light pulse at CT22 (N = 3) in wild-type (black), *Rev-Erbα^−/−^Per1^Brdm1^* (red), *Rev-Erbα^−/−^* (green), and *Per1^Brdm1^* (blue) mice. SCN were collected 45 minutes after the end of the 15-minute light pulse or mock treatment. Values are represented as mean ± SEM. Two-way ANOVA with Bonferroni post-test was performed to determine significant differences between genotypes. Unpaired t-test was used to determine significant differences between light pulsed and dark control animals. * p<0.05; *** p<0.001.

To find a possible explanation for the high amplitude resetting observed in mutants lacking both *Rev-Erbα* and *Per1*, we examined induction of clock genes in response to light. Surprisingly, *Cry1* a clock gene not inducible by light in wild-type mice, was slightly increased in its expression after a light pulse at CT22 in double mutant animals relative to dark controls (Fig. 4A). As expected, wild-type and single mutant mice did not show any *Cry1* induction neither at CT22 nor at CT14 (Fig. 4B). In contrast to CT22, light did not induce *Cry1* expression in *Rev-Erbα^−/−^Per1^Brdm1^* double mutant mice at CT14 (Fig. 4B). In addition, *Per2* was induced to a higher extent in double mutant mice compared to the three other genotypes at CT22 (Fig. 4C) but not at CT14 (data not shown). Expression of *Dec1*, another known light inducible clock gene, was increased stronger in double mutants relative to the other genotypes at CT22 (Fig. 4D) and CT14 (data not shown). The signal intensity for *cFos* was similar between all genotypes after a light pulse at CT22 (Fig. 4E) indicating that the light-signalling pathway is intact. Hence the PRC observed in our double mutants is probably caused by changes in the circadian pacemaker.

To sum up *Rev-Erbα^−/−^Per1^Brdm1^* double mutant mice were fertile and morphologically indistinguishable from wild-type animals. Although they displayed a shorter circadian period in DD, their molecular oscillator seemed to be still functional. However, their response to brief light pulses was altered and they displayed high amplitude resetting. Interestingly, *Cry1* became slightly light inducible if the light signal was presented at the end of the subjective night (CT22).

### Absence of *Cry1* affects suppression of daytime activity in *Rev-Erbα^−/−^Per1^Brdm1^Cry1^−/−^* triple mutant mice

**Figure 5:**
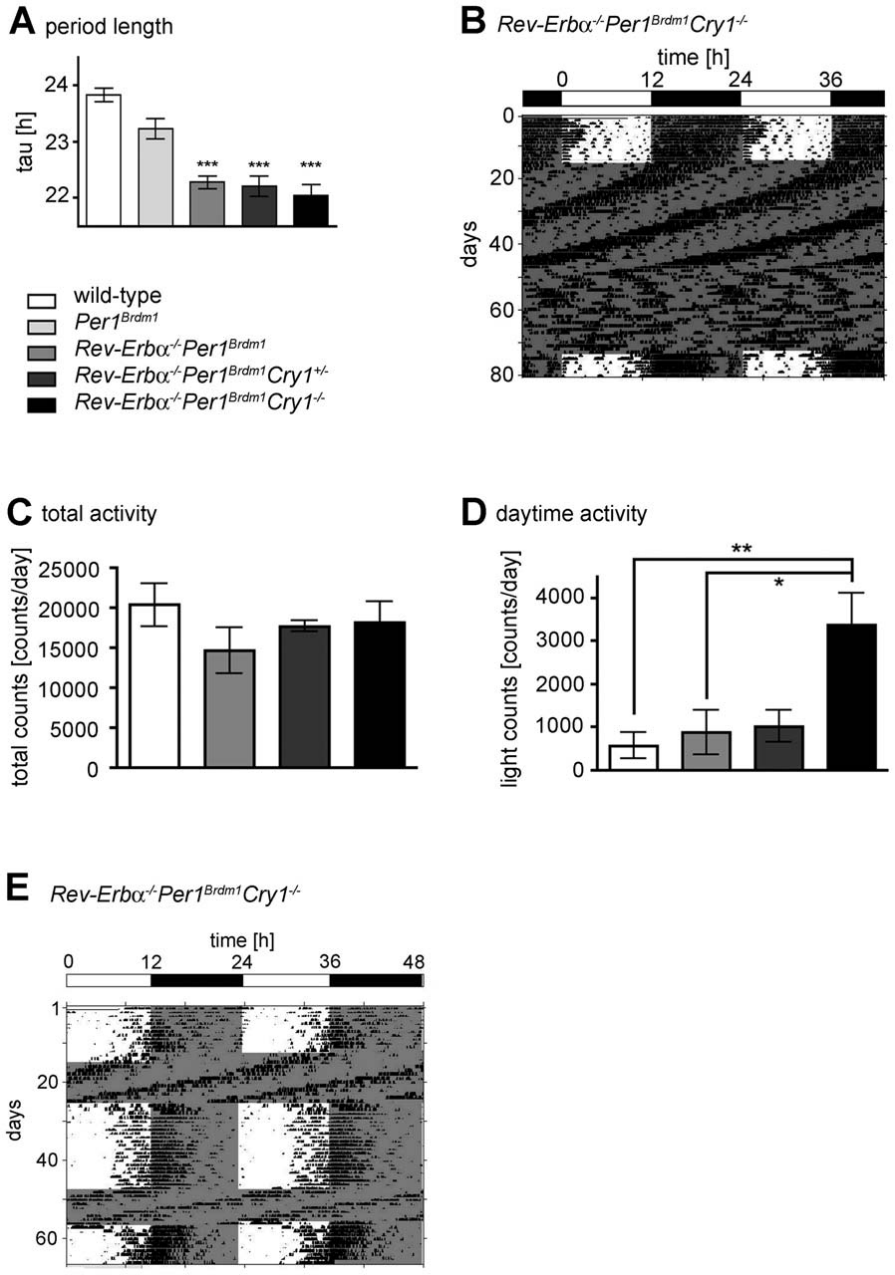
Wheel-running behaviour of *Rev-Erbα^−/−^Per1^Brdm1^Cry1^−/−^* mice. (A) Average free-running period (tau) in constant darkness. Data are represented as mean ± SEM. One-way ANOVA was performed to compare the free-running period length of *Per1^Brdm1^* (23.22±0.19 h; N = 5), *Rev-Erbα^−/−^Per1^Brdm1^* (22.27±0.11 h; N = 16), *Rev-Erbα^−/−^Per1^Brdm1^Cry1^+/−^* (22.20±0.18 h; N = 12) and *Rev-Erbα^−/−^Per1^Brdm1^Cry1^−/−^* (22.04±0.19 h; N = 8) mutant to wild-type mice (23.82±0.12 h; N = 6). *** p<0.001. (B) Arrhythmicity of *Rev-Erbα^−/−^Per1^Brdm1^Cry1^−/−^* triple mutant in constant darkness DD. Locomotor activity record under both light/dark (LD) and DD conditions. Most animals become arrhythmic after about 1 month in DD. Black bars represent wheel-revolutions. The actogram is double-plotted with the activity of the following light/dark cycle plotted to the right and below the previous light/dark cycle. The white and black bar on the top of the actogram indicates light and darkness, respectively. (C) There is no difference in total activity between the genotypes. Data are represented as mean ± SEM. (D) *Rev-Erbα^−/−^Per1^Brdm1^Cry1^−/−^* triple mutant mice display increased daytime activity. Data are represented as mean ± SEM. Unpaired t-test was performed to determine the significant differences between the genotypes. * p = 0.0334, ** p = 0.0087. (E) Masking defect. Typical actogram of a *Rev-Erbα^−/−^Per1^Brdm1^Cry1^−/−^* triple mutant mouse under LD and DD conditions. One can clearly see that the mouse displays increased daytime activity prior to lights off under LD conditions.

In order to unravel whether a change in *Cry1* light induction is related to the observed high amplitude resetting in our double mutant mice, we generated *Rev-Erbα^−/−^Per1^Brdm1^Cry1^−/−^* triple mutant animals (Fig. S4). First, we investigated the circadian phenotype of these mice under both DD and LD conditions. In constant darkness, the circadian period lengths of *Rev-Erbα^−/−^Per1^Brdm1^Cry1^−/−^* (22.04±0.19 h; N = 8), heterozygous *Rev-Erbα^−/−^Per1^Brdm1^Cry1^+/−^* (22.20±0.18 h; N = 12) and *Rev-Erbα^−/−^Per1^Brdm1^* mice (22.27±0.11 h; N = 16) carrying the same genetic background were indistinguishable from each other (Fig. 5A). In contrast to *Rev-Erbα^−/−^Per1^Brdm1^* double mutant animals, triple mutants become arrhythmic after about 1 month in DD (Fig. 5B).

Under LD conditions *Rev-Erbα^−/−^Per1^Brdm1^Cry1^−/−^* triple mutants showed comparable total activity (Fig. 5C) but significantly elevated daytime activity compared to the other genotypes (Fig. 5D). In most cases, daytime activity was clustered before dark onset (Fig. 5E) and in a few mice the dark activity extended into the light phase (Fig. 5B). This indicates reduced masking by light in which *Cry1* may play an important role. This correlates with the observed advance in the phase angle of these animals (Fig. 5E).

Given our findings described above, light resetting was difficult to assess, since the triple mutants become arrhythmic in DD before establishing a stable free running rhythm. Under LD conditions activity onset can't be defined precisely (Fig. 5B, 5E), due to impaired masking. Hence, it is not possible to determine accurately whether the slight light induction of the *Cry1* gene in *Rev-Erbα^−/−^Per1^Brdm1^* mice plays a role in the high amplitude resetting observed in these animals.

## Discussion

Here we show that mice deficient for both *Rev-Erbα^−/−^* and *Per1^Brdm1^* display a dramatically shorter circadian period in DD compared to single mutants and wild-type animals (Fig. 1A–E). As reported previously, *Rev-Erbα^−/−^* and *Per1^Brdm1^* single mutant mice exhibited significantly shorter circadian periods relative to wild-type animals [9], [20]. Hence the shorter period observed in the double mutants might indicate that the concomitant absence of these two clock genes has a cumulative effect on circadian period in DD.

Resetting studies revealed that *Rev-Erbα^−/−^Per1^Brdm1^* double mutants show high amplitude resetting (Fig. 3A–F). A similar phenotype has been reported by Vitaterna et al. [25] for heterozygous *Clock* mutant mice. These mice exhibit a Type 0 PRC in response to 6-h light pulses. Interestingly, light induction of both *Per1* and *Per2* was not affected in the *Clock^+/−^* mutants after 6-hour light exposure. However, basal expression levels of *Per* genes were significantly blunted in both *Clock* homo- and heterozygous mice. In contrast to the observations by Vitaterna *et al.* [25] we could not detect any dampening in *Per2* expression in our animals. *In situ* hybridization profiles for *Per2* were completely identical between *Rev-Erbα^−/−^Per1^Brdm1^* double mutant and wild-type animals (Fig. 2D). Moreover, Vitaterna *et al.* [25] could not detect any differences in light induction of either *Per1* or *Per2* between *Clock* heterozygous and wild-type mice [25]. By contrast, we found that *Per2* induction was stronger in *Rev-Erbα^−/−^Per1^Brdm1^* double mutants compared to wild-types (Fig. 4C). The differences in the two studies might stem from the protocol used for light exposure. Whereas Vitaterna *et al.* [25] used light pulses of 6-hour duration; our protocol is based on short light exposures of 15 minutes. Hence, the mechanisms studied in the two reports are probably different. However, consistent with the results in *Clock* mutants, the *Rev-Erbα^−/−^Per1^Brdm1^* double mutant animals show dampened oscillation of several clock genes in the SCN (Fig. 2) supporting the concept of decreased pacemaker amplitude leading to high amplitude phase shifts.

To test functional consequences of a Type 0-like PRC, we performed jet-lag experiments, one advancing the LD cycle by 8 hours, the other delaying it by 8 hours. We find that *Rev-Erbα^−/−^Per1^Brdm1^* double mutants adapt significantly faster in both paradigms (Fig. 3G, 3H). This is consistent with the findings described above, that the double mutants display larger responses to a light pulse. Hence, larger responses lead to a faster alignment between the clock phase and the LD cycle. Our findings suggest that the actual adaptation to a new time would be fast but is slowed down by components of the circadian clock, which affect various signalling pathways. This is in line with previous findings where it was demonstrated that rapid phase resetting in the liver is counteracted by active signalling pathways [26].

Whether Type 0 or a Type 1 resetting is exhibited in an animal depends not only on species but also on the strength of the stimulus. Increasing the light dose can convert a Type 1 into a Type 0 PRC [27]. In this study we used light pulses of 400 lux and we did not vary intensities. We rather modulated the receiving end of the light input by genetic mutations [27]. However, light seems to reach the SCN in all genotypes, because the immediate early gene *cFos* was induced comparably at CT22 (Fig. 4E). Hence, it is likely, that alterations in core clock components involved in the interpretation of the light signal are the underlying reason for the observed PRC in *Rev-Erbα^−/−^Per1^Brdm1^* double mutants (Fig. 4A, 4C). However, we can't exclude differences between the genotypes in terms of light sensitivity.

Surprisingly, we observed that *Cry1* became slightly light inducible in *Rev-Erbα^−/−^Per1^Brdm1^* double mutant mice at CT22 but not at CT14 (Fig. 4A, 4B). So far, it has never been reported that *Cry1* can be induced by light in mammals [28], [29]. In lower vertebrates like zebrafish (*Danio rerio*), however, *Cry1a* acts as a light-signalling molecule [18]. Its induction as well as that of *Per2* was found to be critical for light-induced phase shifts in this species. Interestingly, we observed an induction of *Per2* in our double mutants at CT22 (Fig. 4C) [30], [31]. Our findings in terms of clock gene induction in the *Rev-Erbα^−/−^Per1^Brdm1^* double mutant mice parallel the ones observed in zebrafish. Interestingly, zebrafish cell lines show high amplitude phase shifts in response to light [18], [32] and zebrafish tissues are directly light responsive [33], [34]. Therefore, we tested whether tissues and cells of *Rev-Erbα^−/−^Per1^Brdm1^* double mutant mice would respond directly to light. However, our experiments indicate that this is not the case (Fig. S5, S6).

Taken together it appears that in the course of evolution complex mechanisms have evolved to adapt land living vertebrates to their environment. In particular, the effects of strong light exposure had to be controlled. This might have led to new functions of *Rev-erbα* and *Per1* keeping behavioral light responses at a moderate level. Our results suggest that loss of *Rev-Erbα* and *Per1* results in a gain of function phenotype in mice and light responses become elevated. This leads to faster adaptation to light stimuli. Under natural conditions (e.g. nocturnal lightning) this would result in jumping of clock phase, which in turn could have adverse effects on the organism. *Rev-Erbα* and *Per1* are probably involved together in attenuating responses to unexpected brief single light pulses to maintain coherence in the circadian system of mammals.

## Materials and Methods

### Animals

All animal work was performed in accordance with the guidelines of the Schweizer Tierschutzgesetz (TSchG, SR455, Abschnitt 2: Art. 5+7, Abschnitt 5: Art. 11 and Abschnitt 6: Art.12–19) and was approved by the state veterinarian of Fribourg (permit FR 101/07) and in accordance with the declaration of Helsinki. Male and female mice were used in this study in equal ratios for each genotype. Animals were raised in LD 12∶12 h and were not in a different cycle before the DD or LL experiments.

The *Rev-Erbα^−/−^Per1^Brdm1^* double mutant mice used in this study were generated by crossing *Rev-Erbα^−/−^* [9] and *Per1^Brdm1^* [20] single mutants. Intercrossing double heterozygous animals produced *Rev-Erbα^−/−^, Per1^Brdm1^, Rev-Erbα^−/−^Per1^Brdm1^* and wild-type animals with a matching genetic background (mixed strain 129Sv/C57Bl/6). Homozygous breeding pairs were established using F2 offspring. To minimize genetic drift, matings were kept together as long as possible. The animals were regularly backcrossed to minimize epigenetic effects.

To obtain *Rev-Erbα^−/−^Per1^Brdm1^Cry1^−/−^* triple mutant mice, animals double mutant for *Rev-Erbα^−/−^Per1^Brdm1^* or *Per1^Brdm1^Cry1^−/−^* [35] were crossed with each other. The *Rev-Erbα^+/−^Per1^Brdm1^Cry1^+/−^* offspring were then intercrossed to obtain the F2 generation. To get triple mutant animals, two mating types were set-up using the F2 mice: in the first case both parents were *Rev-Erbα^−/−^Per1^Brdm1^Cry1^+/−^*, in the second case one parent was *Rev-Erbα^−/−^Per1^Brdm1^Cry1^+/−^* while the other was *Rev-Erbα^+/−^Per1^Brdm1^Cry1^−/−^*. For the experiments we used mice of the F2 (*Per1^Brdm1^, Rev-Erbα^−/−^Per1^Brdm1^, Rev-Erbα^−/−^Per1^Brdm1^Cry1^+/−^*) and F3 (*Rev-Erbα^−/−^Per1^Brdm1^, Rev-Erbα^−/−^Per1^Brdm1^Cry1^+/−^*, *Rev-Erbα^+/−^Per1^Brdm1^Cry1^−/−^*) generation.

The genotype of the offspring was determined by PCR. The PCR protocol for *Rev-Erbα* was according to Preitner *et al*. 2002 [9]. The following primers were used:

*Cry1*_1: 5′-GCA TGA CCC CTC TGT CTG AT-3′

*Cry1*_2: 5′-TGA ATG AAC TGC AGG ACG AG-3′

*Cry1*_3: 5′-AAC ACG CAG ATG CAG TCG-3′

*Per1*_1: 5′-ACA AAC TCA CAG AGC CCA TCC-3′

*Per1_*2: 5′-ATA TTC CTG GTT AGC TGT AGG-3′

*Per1_*3: 5′-CGC ATG CTC CAG ACT GCC TTG-3′

*Rev-Erbα_*1: 5′-CAC CTT ACA CAG TAG CAC CAT GCC ATT CA-3′

*Rev-Erbα_*2: 5′-AAA CCA GGC AAA GCG CCA TTC GCC ATT CA-3′

*Rev-Erbα_*3: 5′-CCA GGA AGT CTA CAA GTG GCC ATG GAA GA-3′

The final primer concentration was 1.0 µM for *Cry1* and 0.5 µM for *Per1* and *Rev-Erbα.* The dNTP (Roche) concentration was 0.4 mM for *Cry1* and 0.2 mM for *Per1* and *Rev-Erbα.* The final MgCl_2_ concentration was 3.0 mM for *Cry1*, 1.5 mM for *Per1* and 3.5 mM for *Rev-Erbα*. To improve annealing, 6 nM (NH_4_)_2_SO_4_ was added to the PCR reaction mix for *Cry1* and *Per1* or 12 nM for *Rev-Erbα.* 2.5 U *Taq* DNA polymerase (Qiagen) were used per 50 µl reaction for *Per1* and 1.25 U per 25 µl reaction for *Rev-Erbα* and *Cry1*. A final concentration of 0.25x and 0.2x Q-solution was used to increase PCR specificity of the *Cry1* and *Per1* reaction, respectively. An initial denaturation was done at 94°C for 2 min. Subsequent denaturation was done at 94°C for 30 s followed by an annealing step of 30 s. The annealing temperature was 56°C for *Per1* and 62°C for *Cry1* and *Rev-Erbα.* For *Per1* and *Rev-Erbα*, the elongation step was performed at 72°C for 1 min. For *Cry1*, the elongation step at 72°C took 7 min. After 34 (*Per1*), 35 (*Cry1*) or 36 (*Rev-Erbα*) cycles, the PCR was ended with a final extension at 72°C for either 7 (*Cry1*) or 10 min (*Per1*, *Rev-Erbα*). From time to time, Southern blot analysis was used to confirm the PCR results obtained for *Per1* [20], [36] and *Cry1* [37].

### Locomotor activity monitoring and circadian phenotype analysis

Mice housing and handling were performed as described earlier [21]. Animals were entrained in LD 12∶12 h for 7–15 days before they were released into DD or LL. Activity was assessed with a running-wheel and evaluated using the ClockLab software package (Actimetrics). Activity records were double plotted in threshold format for 6-min bins. Period length was assessed by χ^2^ periodogram analysis for days 4–10 in DD or LL. To determine light induced phase shifts, an Aschoff Type I protocol was used [22]. Animals were allowed to stabilize their free-running rhythm for at least 2 months prior to the light pulse. The circadian time (CT) at the beginning of the light pulse was calculated for every mouse individually. The phase response curve was established administering 15 min light pulses at CT0 (N [wild-type/*Rev-Erbα^−/−^Per1^Brdm1^*]  = 8/11), CT2 (N = 11/4), CT10 (N = 8/8), CT14 (N = 15/8), CT18 (N = 14/15), CT20 (N = 8/10), and CT22 (N = 13/6).

Mice subjected to experimental jet lag were entrained to LD 12∶12 h. This cycle was then advanced or delayed by 8 hours. Animals were allowed to re-entrain to the new phase for 1 month. The onsets between the two LD phases were determined. The number of days it took the animal to shift from the old onset to the new onset was counted.

### *In situ* hybridization

Locomotor activity was monitored for each mouse to properly determine activity onsets, which is necessary to calculate CT values. To determine circadian gene expression, adult mice were first anesthetized with Attane™ Isoflurane (Provet AG) and then sacrificed on day 6 in DD. For light induction experiments, animals were kept in DD for about 2 months before they were exposed to a 15 min light pulse (400 lux) at different CTs. 45 min after the end of the light pulse, the mice were sacrificed. Control animals were sacrificed without prior light exposure.

Specimen preparation and *in situ* hybridization were carried out as described previously [30]. Briefly, the ^35^S-UTP (1250 Ci/mmol, PerkinElmer) labelled riboprobes were synthesized using the RNAMaxx™ High Yield transcription kit (Stratagene) according to manufacturer's protocol. The *Bmal1* probe was made from a cDNA corresponding to nucleotides (nt) 654–1290 (accession no. AF015953; [38]), *Cry1* corresponds to nt 190–771 (AB000777; [38]), *Cry2* to nt 231–945 (AF156987), *Per2* to nt 229–768 (AF036893; [30]), *cFos* to nt 237–332 [30]. 7 µm thick paraffin sections were dewaxed, rehydrated and fixed in 4% paraformaldehyde. Sections were then permeabilized using a proteinase K (Roche) digestion before they were fixed again and acetylated. After serial dehydration, hybridization was performed over-night at 55°C in a humid chamber. Stringency washes were carried out at 63°C. Slides were subjected to a ribonuclease A (Sigma) digestion and then dehydrated in graded ethanol series. Quantification was performed by densitometric analysis (GS-700 or GS-800, BioRad) of autoradiography films (Amersham Hyperfilm) using the Quantity One software (BioRad). Data from the SCN were normalized subtracting the optical density measured in the lateral hypothalamus next to the SCN. For each experiment at least 3 animals per genotype were used and 4 to 9 adjacent SCN sections per animal were analyzed. Relative RNA abundance values were calculated by defining the highest wild-type mean value of each experiment as 100%. For statistical analysis, all normalized values obtained for one brain were averaged to obtain one final value per animal.

### Statistical analysis

Significant differences were determined using GraphPad Prism 4 software. Depending on the type of data, either unpaired t-test, one- or two-way ANOVA with Bonferroni post-test was performed. Values were considered significantly different with p<0.05 (*), p<0.01 (**), or p<0.001 (***).

For supplemental data see supplemental materials and methods (Methods S1).

## Supporting Information

Methods S1 Supplemental Materials and Methods
(0.04 MB DOC)

Figure S1 PCR analysis of mouse tail DNA for *Rev-Erbα* and *Per1*. Primers for *Rev-Erbα* amplify a fragment of about 340 nucleotides (nt) on the wild-type allele and of about 200 nt on the mutant allele. The PCR for Per1 amplifies a fragment of about 290 nt on the mutant allele and of about 450 nt on the wild-type (wt) allele.
(0.42 MB TIF)

Figure S2 Breeding statistics. (A) Distribution of genotypes in the F2 generation born to *Rev-Erbα+/−Per1+/^Brdm1^* double heterozygous parents. Black bars represent the expected Mendelian distribution while white bars display the observed ratios. The x-axis indicates the genotype of the F2 offspring. The percent of the total mouse number displaying a certain genotype is plotted on the y-axis. (B) Litter frequency of wild-type (white), *Rev-Erbα−/−Per1^Brdm1^* double homozygous (dark grey), and *Rev-Erbα+/−Per1+/^Brdm1^* double heterozygous (light grey) breeding pairs. Data are represented as mean ± SEM. On average, wild-type matings gave birth to a new litter every 32.22±2.06 days (N = 41), *Rev-Erbα−/−Per1^Brdm1^* double homozygous breeding pairs every 32.12±2.33 days (N = 33), and *Rev-Erbα/Per1* double heterozygous couples every 26.42±1.04 days (N = 60). One-way ANOVA with Bonferroni's multiple comparison was performed to compare the litter frequency of the three genotypes. * p<0.05. (C) Number of pups born to wild-type (white), *Rev-Erbα−/−Per1^Brdm1^* double homozygous (dark grey), and *Rev-Erbα+/−Per1+/^Brdm1^* double heterozygous (light grey) breeding pairs. Data are represented as mean ± SEM. On average, wild-type couples gave birth to 6.76±0.51 pups (N = 38), *Rev-Erbα−/−Per1^Brdm1^* double homozygous breeding pairs to 6.18±0.43 pups (N = 33), and *Rev-Erbα/Per1* double heterozygous couples to 6.94±0.31 pups (N = 69). One-way ANOVA with Bonferroni's multiple comparison did not reveal any significant differences in litter size between the three genotypes.
(1.63 MB TIF)

Figure S3 Total and daytime wheel-running activity. (A) Total wheel-running activity of mice kept under LD 12∶12 h (7 days). On average, wild-type mice make 17′738±808 wheel-revolutions per day (N = 32), *Rev-Erbα−/−Per1^Brdm1^* double mutants 16′307±929 (N = 29), *Rev-Erbα−/−* single mutants 14′551±1420 (N = 17), and *Per1^Brdm1^* single mutants 17′718±1195 (N = 16). (B) Rest (rho) phase activity of mice kept under LD 12∶12 h (7 days). Wild-type mice spend 5.98±1.18% (N = 32) of their total activity during the day, *Rev-Erbα−/−Per1^Brdm1^* double mutants 6.74±1.4% (N = 29), *Rev-Erbα−/−* single mutants 4.6±1.4% (N = 17), and *Per1^Brdm1^* single mutants 6.76±2.3% (N = 16). Data are represented as mean ± SEM. One-way ANOVA with Bonferroni's multiple comparison did not reveal any significant difference between genotypes for both total and rho phase activity. (C) 24-h average distribution of activity for wild-type (black; N = 32), *Rev-Erbα−/−Per1^Brdm1^* double mutant (red; N = 29), Rev-Erbα−/− single mutant (green; N = 17), and *Per1^Brdm1^* single mutant mice (blue; N = 15) kept in LD 12∶12 h. The black and white bar on the bottom depicts the light and dark phase, respectively. Data are represented as mean only. The x-axis indicates the phase angle while the y-axis displays the activity as average counts/minute over a ten-minute interval. Statistical analysis did not reveal any difference between the genotypes.
(4.80 MB TIF)

Figure S4 Characterization of *Rev-Erbα−/−Per1^Brdm1^Cry1−/−* triple mutant mice by PCR. PCR analysis of mouse tail DNA for Cry1. Fragments of 2.3 and 3.1 kbp were amplified on the wild-type and mutant allele, respectively.
(2.65 MB TIF)

Figure S5 Ear cells of *Rev-Erbα−/−Per1^Brdm1^* double mutant mice are not directly light responsive. (A) *Cry1* and (B) *Per2* induction in the ear after a light pulse at ZT22 measured in wild-type (black), *Rev-Erbα−/−Per1^Brdm1^* (red), *Rev-Erbα−/−* (green), and *Per1^Brdm1^* (blue) mice. Mice were sacrificed at ZT22. The ears were kept in cell culture medium and immediately subjected to a 30 min light pulse. Another 30 min later the tissue was harvested for further analysis. Values are represented as mean ± SEM (N = 3). Two-way ANOVA with Bonferroni post-test did not detect significant differences between genotypes.
(0.77 MB TIF)

Figure S6 Mouse dermal fibroblasts of *Rev-Erbα−/−Per1^Brdm1^* double mutant mice are not directly light responsive. Immortalized MDFs were subjected to a 30 min light pulse. A UV-filter was between the cells and the light source. The induction of *Cry1* (A, B), *Per2* (C, D) and *Bmal1* (E, F) were assessed using real-time PCR during 8 h. *Rev-Erbα−/−Per1^Brdm1^* double mutant MDFs are plotted on the left side (A, C, E) while the wild-type cells are on the right side (B, D, F). Data are represented as mean ± SD (N = 2 except for wild-type time-point 0 where N = 1). Two-way ANOVA with Bonferroni post-test did not reveal any significant differences between the light pulse samples and the dark controls. Values were normalized to the highest mean level within an experiment.
(1.34 MB TIF)
