# Supplementary material for: High Amplitude Phase Resetting in Rev-Erbα/Per1 Double Mutant Mice
Source: PLoS One. 2010 Sep 2;5(9):e12540. doi: 10.1371/journal.pone.0012540 (PMC2932729; doi:10.1371/journal.pone.0012540)
Supplement: Methods S1 — Supplemental Materials and Methods (0.04 MB DOC) [file pone.0012540.s001.doc]

**Supplemental data**

**Supplemental methods**

**Preparation of mouse dermal fibroblasts (MDFs)**

To generate MDFs, mice were anesthetized with isoflurane (Provet) and sacrificed by cervical dislocation. The head of the mouse was sprayed with 70 % ethanol before the top parts of the ears were cut and put into ice-cold DMEM high glucose (4.5 g/l) medium (Amimed) supplemented with 1 mg/ml collagenase type I (Sigma), 5 μg/ml amphotericine B (Sigma), 500 U/ml penicillin/streptomycin (Amimed). Under the laminar flow hood the ears were cut into small pieces with a scalpel blade and were incubated over night at 37°C and 5 % CO_2_. The next day, the cell suspension was passed repeatedly through a narrowed sterile glass Pasteur pipette before cells were spun down for 10 min at 4000 rpm. The MDFs were resuspended in DMEM high glucose medium supplemented with 20 % heat inactivated (30 min at 56°C) fetal calf serum (HIFCS, Amimed), 1 % penicillin/streptomycin, 1 % L-glutamine (Amimed) before they were seeded on a 6-well plate. Once they were confluent, cells were split using 0.25 % trypsine/1 mM EDTA (Gibco) and diluted in DMEM high glucose medium supplemented with 20 % HIFCS, 1 % penicillin/streptomycin and 1 % glutamine during three passages. Then the cells were transferred to a 10 cm dish and the HIFCS was reduced to 10 %. Since primary MDFs reach their senescence after a limited number of population doublings, they were immortalized. MDFs immortalize spontaneously at low frequencies by passing through replicative senescence after around 20 passages thanks to random genetic mutations. Cells were kept in liquid nitrogen until use.

**Light pulse experiment on MDFs**

Wild-type and *Rev-Erbα*^-/-^*Per1^Brdm1^* MDFs were synchronized by addition of DMEM containing 100 nM dexamethasone (Sigma) (Balsalobre et al., 2000). After 20 min, the cells were washed 2 times with serum-free DMEM before the medium was changed to phenol red-free DMEM (Amimed) supplemented with 10 % HIFCS. A baseline experiment revealed that in both wild-type and mutant MDFs *Per2* and *Cry1* expression were low 14 h after the dexamethasone shock (samples were taken every 4 h during 56 h). Hence, we decided to administer a 30 min light pulse at this time point. Samples were taken after 0, 1, 2, 3, 4, 5, 6, and 8 h after the start of the light pulse. At every time point, a dark control was taken.

**Light pulse experiment on mouse ears**

Two 12-well plates (Falcon) were prepared containing 4 ml of 37°C warm transparent DMEM high glucose medium (Gibco) supplemented with 1 % penicillin/streptomycin (Amimed) per well. This medium contains 25 mM HEPES which is important for the pH. To exclude any influence of light on the mice and the ears all manipulations were performed wearing night-vision goggles (Rigel 3200). The animals were anesthetized with isoflurane (Provet) before they were sacrificed at ZT22 (± 15 min) by cervical dislocation. The top part of the ears were cut and put into the medium. One ear per mouse was exposed to the light pulse while the other ear of the same mouse served as a dark control. Three animals were sacrificed per genotype. Once all the wells were filled with tissue, one plate was wrapped with aluminium foil while the other was not. Then both plates were transferred into a 37°C water bath above which a light bulb was installed (Mazdafluor, Symphony, Azura 965, SF 18W/AZU). 30 min after the end of the 30 min light pulse the ears were frozen in liquid nitrogen.

**RNA isolation, reverse transcription and real-time PCR**

Total RNA was extracted from cells using the Absolutely RNA nanoprep Kit (Stratagene) according to the manufacturer’s instructions. The defrozen ears were cut into small pieces using a scalpel blade before they were homogenized in 800 μl of RNAbee (Tel-Test; AMS Biotechnology) using a polytron (IKA). Total RNA was extracted according to the manufacturer’s instructions. cDNA was synthesized using random hexamer primers (Roche) and SuperScript II (Invitrogen) according to manufacturer’s instructions. Real-time PCR was performed as described earlier (Preitner *et al.*, 2002). In brief, the amplification was carried out on a BioRad iCycler at 60°C for 1 min followed by a denaturation at 94°C for 15 sec. After 45 cycles ΔC_T_ was determined. All samples were normalized to *Gapdh*. Relative mRNA levels were assessed by defining the mean value obtained from light-pulsed wild-type ears as 100 %. The following primers and probes at a final concentration of 300 nM and 200 nM were used, respectively:

*mBmal1* fw: 5’-CCA AGA AAG TAT GGA CAC AGA CAA A-3’

*mBmal1* rv: 5’-GCA TTC TTG ATC CTT CCT TGG T-3’

*mBmal1* taq: 5’-TGA CCC TCA TGG AAG GTT AGA ATA TGC AGA A-3’

*mCry1* fw: 5’-CTG GCG TGG AAG TCA TCG T-3’

*mCry1* rv: 5’-CTG TCC GCC ATT GAG TTC TAT G-3’

*mCry1* taq: 5’-CGC ATT TCA CAT ACA CTG TAT GAC CTG GAC A-3’

*mPer2* fw: 5’-ATG CTC GCC ATC CAC AAG-3’

*mPer2* rv: 5’-TGT GAT GTA CTC CCC GTT GC-3’

*mPer2* taq: 5’-ATC CTA CAG GCC GGT GGA CAG CC-3’

*mGapdh* fw: 5’-CAT GGC CTT CCG TGT TCC TA-3’

*mGapdh* rv: 5’-CCT GCT TCA CCA CCT TCT TGA-3’

*mGapdh* taq: 5’-CCG CCT GGA GAA ACC TGC CAA GTA TG-3’
