# Supplementary figures and images for: High Amplitude Phase Resetting in Rev-Erbα/Per1 Double Mutant Mice
Source: PLoS One. 2010 Sep 2;5(9):e12540. doi: 10.1371/journal.pone.0012540 (PMC2932729; doi:10.1371/journal.pone.0012540)

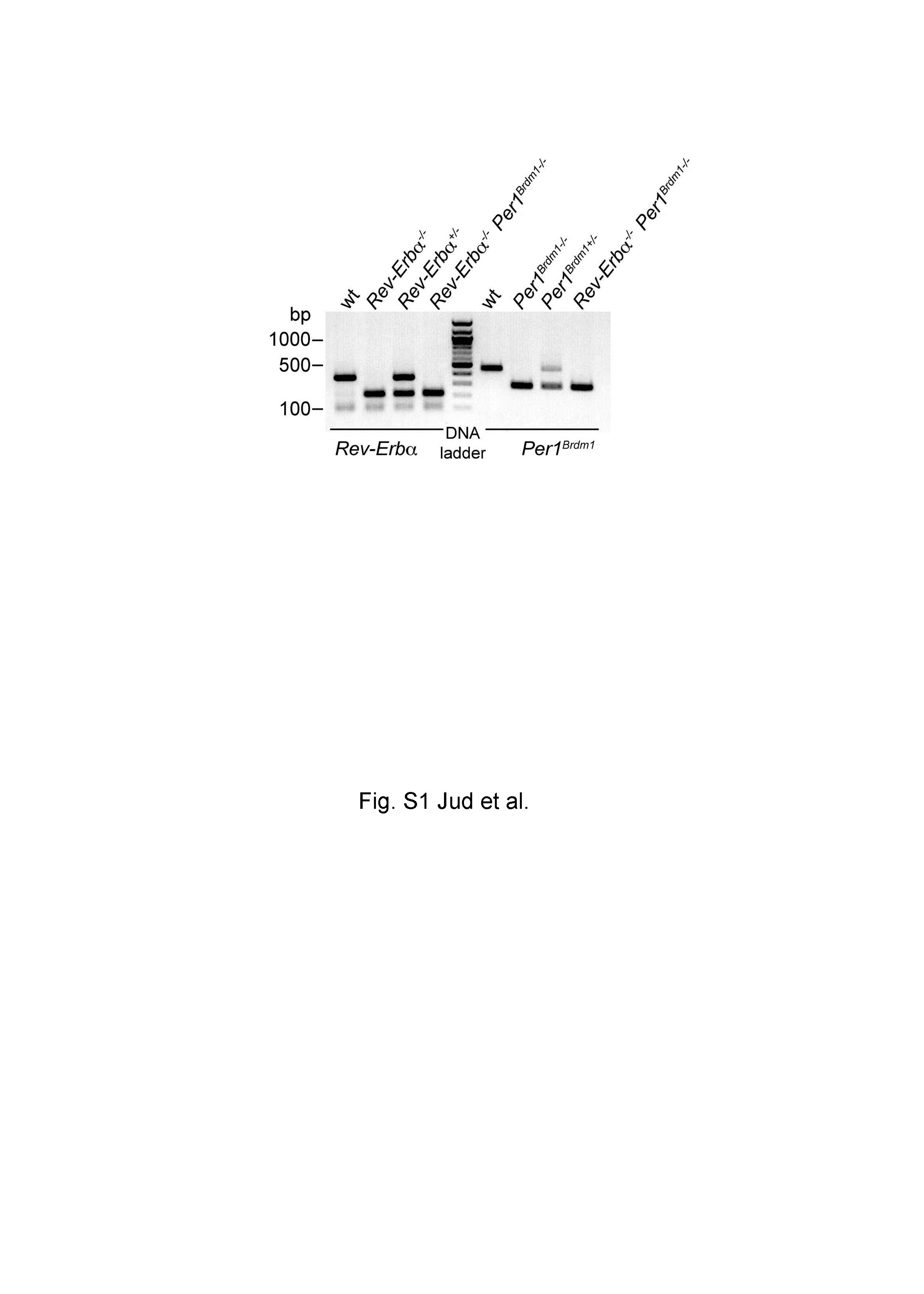

Supplement: Figure S1 — PCR analysis of mouse tail DNA for Rev-Erbα and Per1. Primers for Rev-Erbα amplify a fragment of about 340 nucleotides (nt) on the wild-type allele and of about 200 nt on the mutant allele. The PCR for Per1 amplifies a fragment of about 290 nt on the mutant allele and of about 450 nt on the wild-type (wt) allele. (0.42 MB TIF) [file pone.0012540.s002.tif]

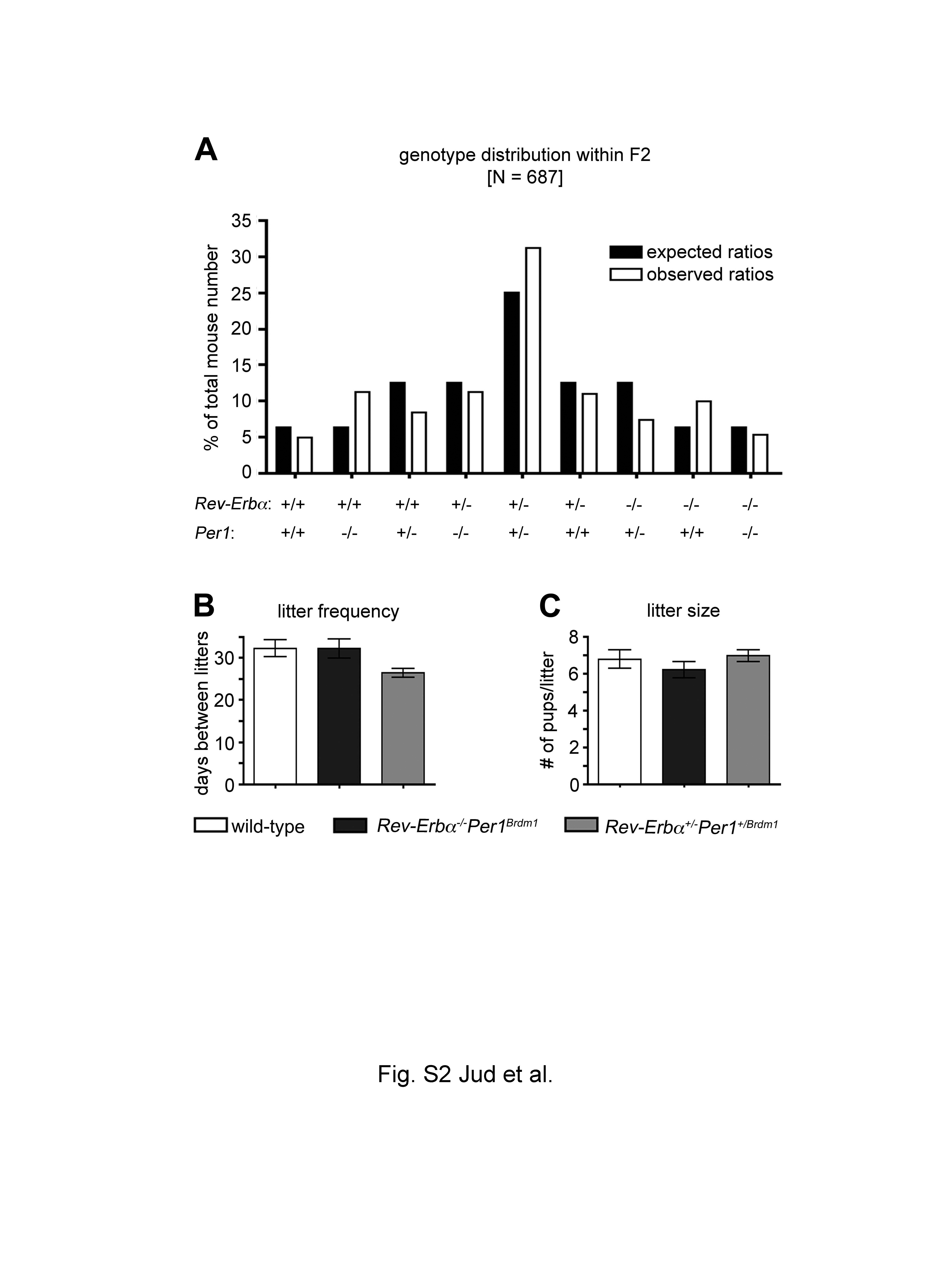

Supplement: Figure S2 — Breeding statistics. (A) Distribution of genotypes in the F2 generation born to Rev-Erbα+/−Per1+/Brdm1 double heterozygous parents. Black bars represent the expected Mendelian distribution while white bars display the observed ratios. The x-axis indicates the genotype of the F2 offspring. The percent of the total mouse number displaying a certain genotype is plotted on the y-axis. (B) Litter frequency of wild-type (white), Rev-Erbα−/−Per1Brdm1 double homozygous (dark grey), and Rev-Erbα+/−Per1+/Brdm1 double heterozygous (light grey) breeding pairs. Data are represented as mean ± SEM. On average, wild-type matings gave birth to a new litter every 32.22±2.06 days (N = 41), Rev-Erbα−/−Per1Brdm1 double homozygous breeding pairs every 32.12±2.33 days (N = 33), and Rev-Erbα/Per1 double heterozygous couples every 26.42±1.04 days (N = 60). One-way ANOVA with Bonferroni's multiple comparison was performed to compare the litter frequency of the three genotypes. * p<0.05. (C) Number of pups born to wild-type (white), Rev-Erbα−/−Per1Brdm1 double homozygous (dark grey), and Rev-Erbα+/−Per1+/Brdm1 double heterozygous (light grey) breeding pairs. Data are represented as mean ± SEM. On average, wild-type couples gave birth to 6.76±0.51 pups (N = 38), Rev-Erbα−/−Per1Brdm1 double homozygous breeding pairs to 6.18±0.43 pups (N = 33), and Rev-Erbα/Per1 double heterozygous couples to 6.94±0.31 pups (N = 69). One-way ANOVA with Bonferroni's multiple comparison did not reveal any significant differences in litter size between the three genotypes. (1.63 MB TIF) [file pone.0012540.s003.tif]

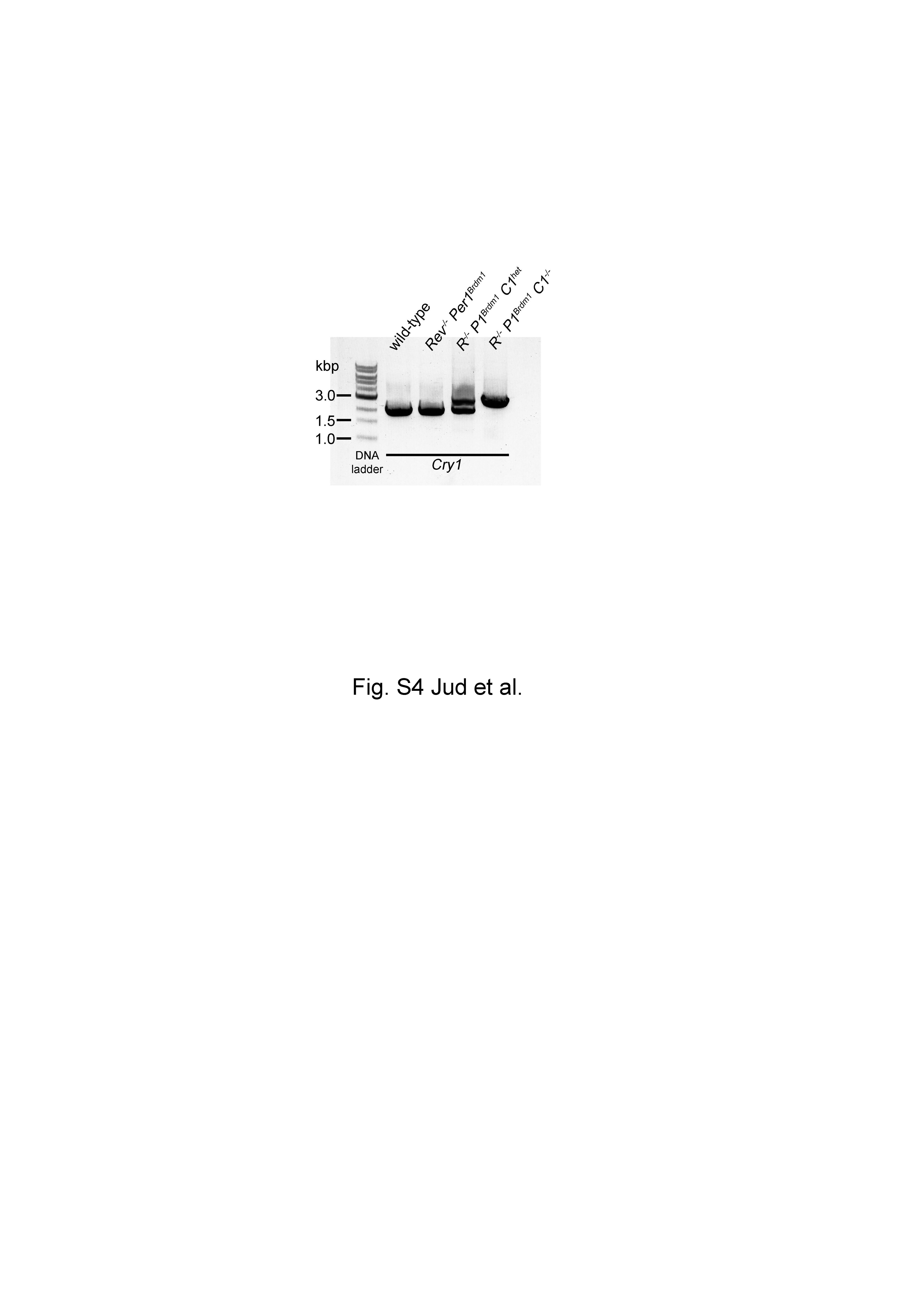

Supplement: Figure S4 — Characterization of Rev-Erbα−/−Per1Brdm1Cry1−/− triple mutant mice by PCR. PCR analysis of mouse tail DNA for Cry1. Fragments of 2.3 and 3.1 kbp were amplified on the wild-type and mutant allele, respectively. (2.65 MB TIF) [file pone.0012540.s005.tif]
